# Gastroesophageal reflux disease and chronic cough: A possible mechanism elucidated by ambulatory pH‐impedance‐pressure monitoring

**DOI:** 10.1111/nmo.13707

**Published:** 2019-09-03

**Authors:** Xiaoqing Li, Sihui Lin, Zhifeng Wang, Hong Zhang, Xiaohong Sun, Ji Li, Dong Wu, Meiyun Ke, Xiucai Fang

**Affiliations:** ^1^ Departement of Gastroenterology Peking Union Medical College Hospital, Chinese Academy of Medical Sciences and Peking Union Medical College Beijing China; ^2^ Department of Gastroenterology The First Affiliated Hospital of Xiamen University Xiamen China; ^3^ Department of Respiration Peking Union Medical College Hospital, Chinese Academy of Medical Sciences and Peking Union Medical College Beijing China

**Keywords:** ambulatory pH‐impedance‐pressure monitoring, chronic cough, esophageal motility, exposure to acid, gastroesophageal reflux disease

## Abstract

**Background:**

The pathophysiological mechanism(s) of gastroesophageal reflux disease (GERD)‐related chronic cough (CC) is unclear. We aimed to determine the mechanism of reflux‐induced cough by synchronous monitoring of reflux episodes, esophageal motility, and cough.

**Methods:**

Patients with GERD were prospectively enrolled and classified into GERD with CC (GERD‐CC) and without CC (GERD) groups. Twenty‐four‐hour ambulatory pH‐impedance‐pressure monitoring was performed; the reflux patterns, esophageal motility during prolonged exposure to acid and characteristics of reflux episodes that induced coughing paroxysms were analyzed.

**Key Results:**

Thirty‐one patients with GERD‐CC and 47 with GERD were enrolled; all of whose monitoring results fulfilled the criteria for diagnosis of GERD. Patients with GERD‐CC had higher reflux symptom scores, longer exposure to acid, higher DeMeester scores, and more frequent reflux episodes, proximal extent reflux detected by impedance, and higher percentage of strongly acidic reflux than patients in the GERD group (all *P* < .05). Of 63 reflux‐cough episodes identified in the GERD‐CC group, 74.6% of distal reflux and 67.0% of proximal reflux episodes were acidic. More patients had low pan‐esophageal pressure in primary peristalsis (48.5% vs 11.8%, *P* = .000) and synchronous contraction in secondary peristalsis during prolonged exposure to acid in the GERD‐CC than in the GERD group (63.9% vs 9.1%, *P* = .000).

**Conclusions & Inferences:**

Proximal acidic reflux and distal reflux‐reflex are jointly associated with reflux‐induced cough in patients with GERD. Low pan‐esophageal pressure in primary peristalsis and synchronous contraction in secondary peristalsis may play important roles in GERD‐associated chronic cough.

## Graphical abstract

Proximal acidic reflux and distal reflux‐reflex, as well as ineffective esophageal motility during prolonged acid exposure, were associated with GERD‐associated chronic cough. Optimization of proton pump inhibitor and/or addition of prokinetic therapy might enhance the treatment in GERD patients with chronic cough.

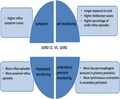

**Abbreviations**
ACEI: angiotensin‐converting enzyme inhibitors
AET: acid exposure time
BAL: bronchoalveolar lavage
CC: chronic cough
EE: erosive esophagitis
GER: gastroesophageal reflux
GERD: gastroesophageal reflux disease
HRM: high‐resolution manometry
IEM: ineffective esophageal motility
IQRs: interquartile ranges
LA: Los Angeles
LES: lower esophageal sphincter
NERD: non‐erosive reflux disease
SAP: symptom association probability
SD: standard deviation

**Key Points**

- The hypotheses of reflux and reflex have been proposed for explaining chronic cough with gastroesophageal reflux disease (GERD).
- Patients with GERD and chronic cough have more severe reflux episodes and proximal extent reflux than those without chronic cough; thus, both proximal acidic reflux and distal reflux‐reflex are associated with reflux‐associated cough.
- Low pan‐esophageal pressure in primary peristalsis and synchronous contraction in secondary peristalsis during prolonged exposure to acid may play an important role in GERD‐associated chronic cough.

## 1 INTRODUCTION

According to the Montreal definition and classification, gastroesophageal reflux disease (GERD) causes esophageal and extra‐esophageal symptoms.^1^ Chronic cough (CC), defined as cough for more than 8 weeks, is accepted as a definite extra‐esophageal symptom and affects estimated 9%‐33% of European and US individuals with GERD.^2^ GERD, asthma, and postnasal drip are considered as the most important factors contributing to chronic cough. Two possible pathophysiological mechanisms, namely “reflux theory” and “reflex theory,” may cause reflux‐induced cough episodes.^3^

Acid reflux has been identified as one of the most important causes of chronic cough and some patients with CC benefit from anti‐acid therapy.^4^, ^5^ However, many patients experience significant adverse effects of anti‐acid therapy without benefit. Studies have also shown that weakly acidic reflux plays a role in reflux‐induced cough.^6^, ^7^ Increased esophageal exposure to acid associated with esophageal dysmotility has been identified in patients with extra‐esophageal symptoms.^8^, ^9^, ^10^ Most studies using traditional manometric techniques have identified associations between ineffective esophageal motility (IEM) and both long exposure time and poor clearance of reflux events. The current Chicago Classification^11^ and Lyon Consensus^12^ listed more details regarding esophageal dysmotility as assessed by esophageal high‐resolution manometry (HRM); however, HRM has infrequently been used to assess GERD with CC.

An esophageal‐tracheobronchial reflex mediated by afferent nerves in the distal esophagus, defined as reflex theory, is another possible explanation for the relationship between GERD and CC. Enhanced cough reflex sensitivity and neurogenic airway inflammation are associated with reflux‐induced cough.^13^, ^14^, ^15^ However, few ambulatory data have been published.

Accurate diagnosis of reflux‐associated CC is challenging, one problem being identification of the refluxate's properties. Esophageal pH monitoring does not detect all gastroesophageal events, particularly when the refluxate is weakly acidic or non‐acidic. Combined esophageal pH‐impedance monitoring is a new means of detecting and classifying reflux events into acidic, weakly acidic, and non‐acidic reflux.^16^ Another unresolved problem is establishment of a causal relation between reflux and cough. The most frequently used indicator is symptom association probability (SAP),^17^ which indicates whether or not the relationship between reflux and perceived symptoms is random.^18^ However, the recorded time of cough may not coincide exactly with when it occurred and symptoms occurring during sleep may be omitted. Therefore, a new objective method for recording cough is needed. Ambulatory pH‐impedance‐pressure monitoring has been used in a few studies to record occurrence of cough, a 2‐minute time window being used to assess the temporal association between reflux and cough in patients with unexplained CC.^19^, ^20^, ^21^, ^22^ However, there are still several unanswered questions, such as why some patients with GERD have chronic cough while others do not and whether esophageal dysmotility plays a crucial role in inducing reflux‐associated cough.

Therefore, the aims of our study were to (a) compare the reflux characteristics of patients of GERD with and without CC; (b) identify ambulatory esophageal motility changes around reflux and coughing paroxysms; and (c) identify a subset of patients with GERD and CC who might benefit from anti‐acids and/or prokinetics.

## 2 MATERIALS AND METHODS

### 2.1 Participants

Consecutive patients (18‐65 years old) with typical reflux symptoms (heartburn and/or regurgitation) for more than 3 months were enrolled in a tertiary gastroenterology clinic in Peking Union Medical College Hospital, China, from October 2014 to October 2015. All patients had experienced mild reflux symptoms for at least 2 days per week or moderate/severe reflux symptoms for more than 1 day per week during the previous month. The frequency and degree of reflux symptoms were recorded and reflux symptom scores were used to assess severity of reflux symptom. Patients with peptic ulcer, malignancy, severe systemic disease, history of upper gastrointestinal surgery, or pregnancy were excluded from the study.

The typical GERD patients were then divided into two subgroups according to whether they had CC during the course of GERD: a GERD with CC (GERD‐CC group) and a GERD without CC (GERD group). CC was defined as cough persisting for more than 8 weeks, excluding asthma, postnasal drip, or use of angiotensin‐converting enzyme inhibitors (ACEIs).

After enrollment, all participants underwent gastroscopy unless they had undergone gastroscopy within the previous month. They were diagnosed as having either erosive esophagitis (EE), which was classified according to the Los Angeles (LA) classification or non‐erosive reflux disease (NERD), that is, without esophageal mucosal erosions.

All participants provided written informed consent. This study was approved by the Ethics Committee of Peking Union Medical College Hospital (No. JS‐829).

### 2.2 Ambulatory pH‐impedance‐pressure monitoring

**Figure 1:**
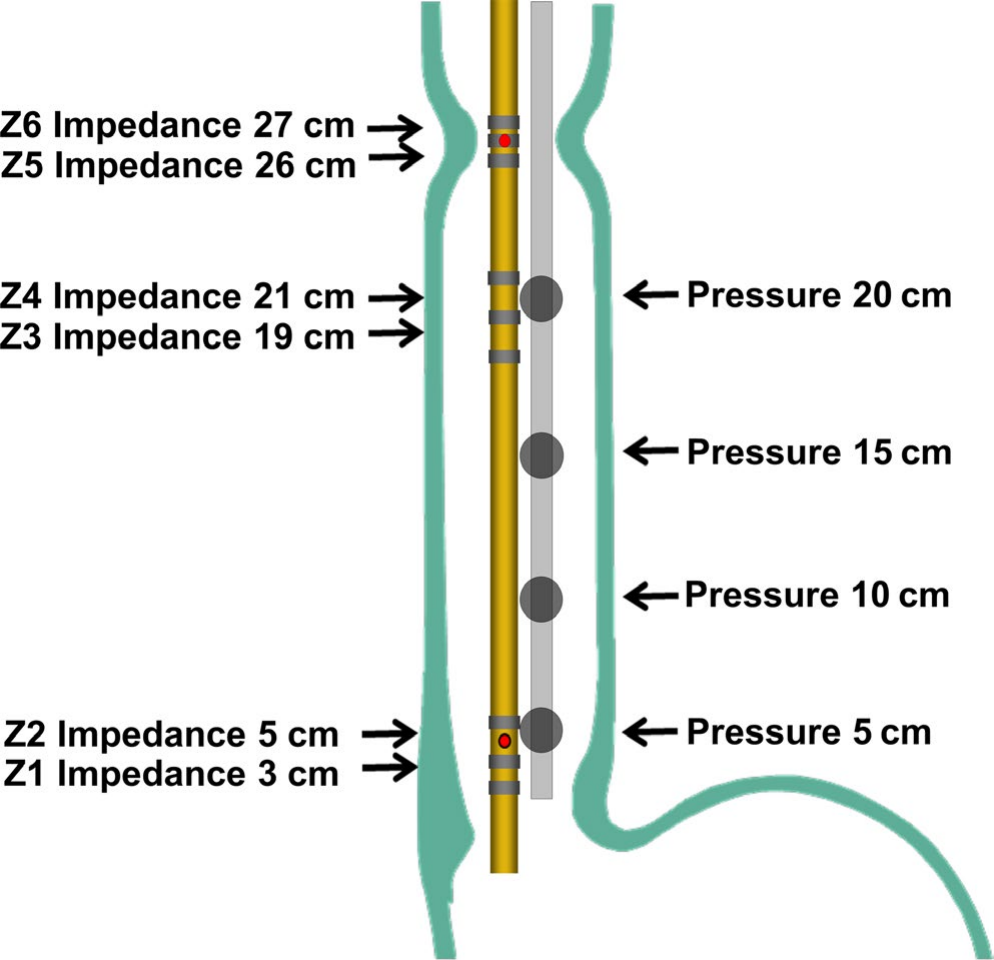
Positions of electrodes of the ambulatory pH‐impedance‐pressure monitoring system. Red spot: pH electrodes; grey loop: impedance electrodes; black spot: pressure sensors. The values in the figure refer to the distance from the upper border of the lower esophageal sphincter

All participants underwent 24‐hour ambulatory pH‐impedance‐pressure monitoring, which was achieved through two catheters, a combined pH‐impedance catheter and an intra‐esophageal pressure catheter. The pH‐impedance catheter (6.9 French, MMS‐Z2L‐A‐LES; MMS, An Enschede, the Netherlands) has six impedance channels and two pH antimony electrodes (positioned at 5 cm [pH1] and 27 cm [pH2] above the lower esophageal sphincter [LES]). The intra‐esophageal pressure catheter (GIM6000; MMS) has four pressure sensors, which are located 5 cm (P1), 10 cm (P2), 15 cm (P3), and 20 cm (P4) proximal to the upper border of the LES. The positions of pH‐impedance‐pressure electrodes were shown in Figure 1. Both catheters were introduced simultaneously via the same nostril and taped to the face. The pH, impedance, and pressure signals were stored on a digital data logger (Omega; MMC). Monitoring was performed for 24 hours.

Each participant was supplied with three standard nutritious test meals by the diet center of our hospital. During the 24‐hours of measurement, only the test meals and water were consumed. The participants were instructed to note down times of reflux symptoms, meals, and sleep and were encouraged to maintain their normal activities throughout recording. All participants tolerated and completed the measurements.

### 2.3 Data analysis

#### 2.3.1 Assessment of reflux symptoms

Reflux symptoms during the previous month were scored as described by Vigneri et al^23^ as follows: reflux symptom score = severity score × frequency score; separate scores for heartburn and acid reflux are added together. The severity of each symptom is graded as follows: 0 = no symptoms; 1 = mild symptoms with spontaneous remission and no interference with normal activity or sleep; 2 = moderate symptoms with spontaneous but slow remission and mild interference with normal daily activities or sleep; and 3 = severe symptoms without spontaneous remission and marked interference with normal daily activities or sleep. The frequency of each symptom was scored as follows: 0 = none, 1 = <2 days per week, 2 = 2‐4 days per week, and 3 = >4 days per week.

#### 2.3.2 Reflux variables

Gastroesophageal reflux (GER) variables were analyzed by an MMS Solar GI acquisition system from recorded pH‐impedance data. Esophageal acid reflux was defined as pH < 4 and expressed as acid exposure time (AET) in minutes and durations of acid reflux episodes. Acid exposure lasting ≥5 minute was defined as prolonged acid reflux. Severity of acid reflux was expressed as DeMeester scores.^24^ In accordance with the Lyon Consensus,^12^ AET >6% or more than 80 impedance‐detected reflux episodes was considered conclusive evidence for pathologic reflux.

Impedance‐detected reflux was classified on the basis of pH monitoring data as acidic reflux when pH < 4, weakly acid reflux when pH 4‐7, and non‐acidic reflux when pH > 7. Distal reflux was defined as reflux limited to within 19 cm of the LES, proximal reflux as reflux reaching further than 19 cm from the LES, and high reflux as reflux reaching further than 26 cm from the LES.

#### 2.3.3 Esophageal motility and cough

Esophageal peristalsis was identified as primary or secondary peristalsis according to its association with swallowing during ambulatory pressure monitoring. Primary peristalsis is peristalsis induced by swallowing and transporting of boluses inside the esophagus, whereas secondary peristalsis is triggered by various intra‐esophageal stimuli (ie, refluxate in this study) in the absence of swallowing.^25^, ^26^ In this study, we identified those peristalsis as primary in which the bolus entry at each specific level obtained at the 50% point between 3 seconds pre‐swallow impedance baseline and impedance nadir during bolus presence and bolus exit determined as return to this 50% point on the impedance‐recovery curve.^27^ We detected swallows to distinguish primary from secondary peristalsis by deglutitive impedance gradient and impedance traces. There was no impedance change at the most proximal impedance electrode when secondary peristalsis happened. We analyzed primary and secondary peristalsis only during the longest period of acid reflux in each patient.

Further, ineffective peristalsis,^28^, ^29^ also called ineffective esophageal motility (IEM), was defined as contraction amplitude of P1 and P2 in the proximal esophagus <12 mm Hg or that of P3 and P4 in the distal esophagus <25 mm Hg, or antiperistalsis or synchronous contraction occurred in two or more channels during ambulatory pressure monitoring.

A cough was defined as a rapid, short duration, simultaneous pressure peak with time to peak <1 second^19^, ^21^ and with the same pressure configuration at all intra‐esophageal recording sites on ambulatory esophageal manometry. A coughing paroxysm was defined as two or more rapid simultaneous pressure peaks within 3 seconds. Only coughing paroxysms were analyzed in this study.

#### 2.3.4 Association between reflux and coughing paroxysms

**Figure 2:**
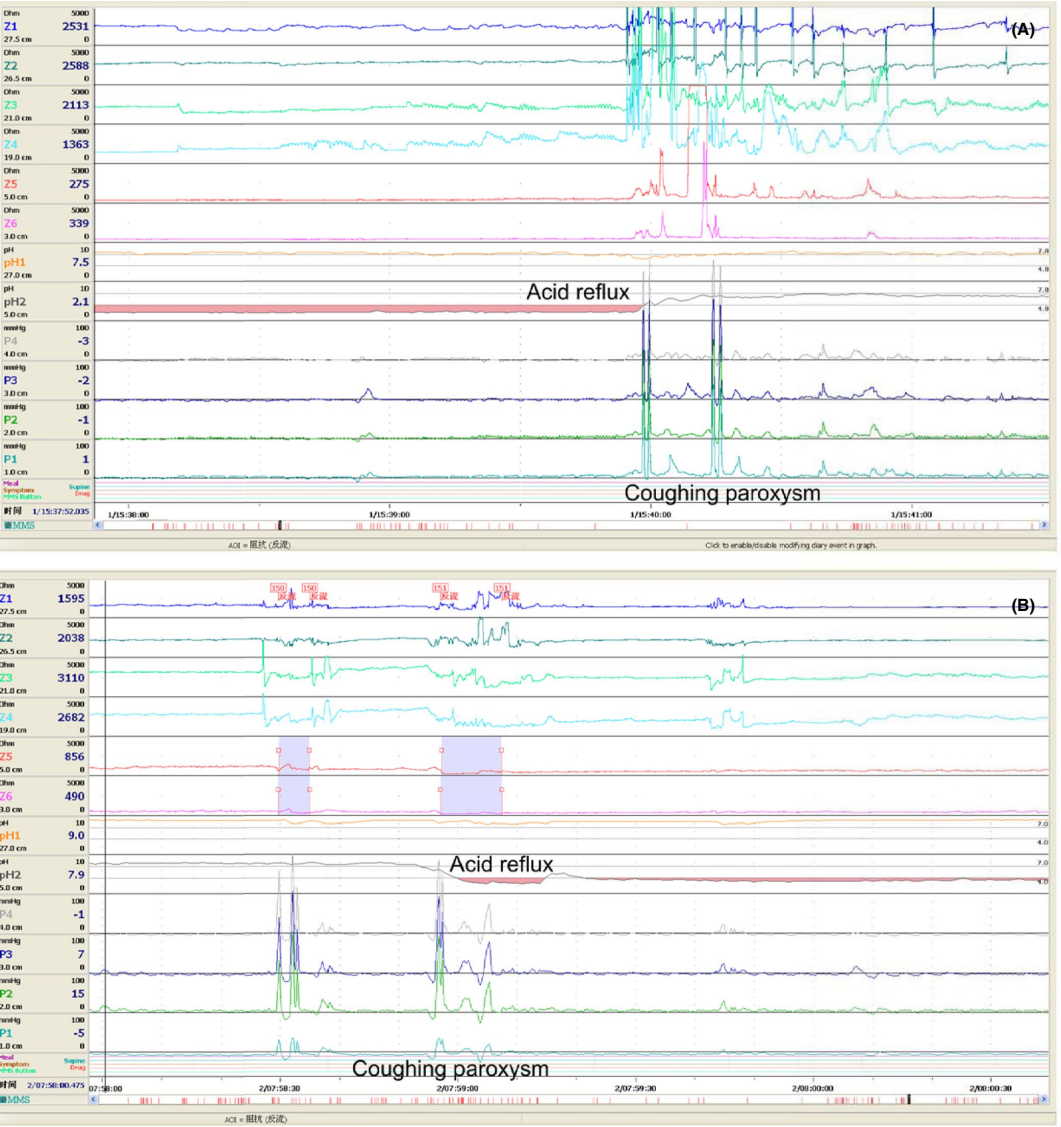
Tracings of ambulatory esophageal pH‐impedance‐pressure monitoring revealing a temporal correlation with reflux‐coughing paroxysms. A, Reflux‐cough episode; B, Cough‐reflux episode

Coughing paroxysms were considered related to a reflux episode if they occurred within 2 minutes of a reflux episode.^19^, ^20^ Coughing paroxysms within 2 minutes after the onset of a reflux episode were considered reflux‐cough episodes (Figure 2A). Reflux episodes occurring within 2 minutes after a coughing paroxysm were defined as cough‐reflux episodes (Figure 2B). When there was more than 2 minutes between a reflux episode and coughing paroxysm, they were defined as being unrelated.

### 2.4 Statistical analysis

SPSS 18.0 (IBM) was used for statistical analysis of data. Parametric and non‐parametric data are presented as the mean ± standard deviation (SD) and median and interquartile ranges (IQRs), respectively. Normally distributed continuous variables were compared using paired samples t tests and non‐normally distributed data using Mann–Whitney *U* tests. The chi‐square test was used for categorical variables. *P* < .05 was considered to denote statistical significance.

## 3 RESULTS

### 3.1 Patient cohort

The study cohort comprised 78 patients with GERD, 31 of whom had CC and were accordingly assigned to the GERD‐CC group, the remaining 47 (without CC) being assigned to the GERD group. Relevant patient characteristics were shown in Table 1. There were no significant differences in gender, age, bodyweight, height, and body mass index between the two groups.

**Table 1:** Relevant patient characteristics

	GERD‐CC group (n = 31)	GERD group (n = 47)	*P *value
Female (n, [%])	17 (54.8%)	24 (51.1%)	.74
Age (y)	52.1 ± 10.8	51.1 ± 12.0	.70
Body weight (Kg)	66.7 ± 10.2	66.6 ± 14.3	.88
Height (cm)	165.9 ± 8.6	165.5 ± 7.1	.84
BMI (Kg/m^2^)	24.2 ± 3.0	24.0 ± 4.2	.87

Note

Data are presented as the mean ± SD or number (percentage).

Abbreviations: BMI, body mass index; GERD, gastroesophageal reflux disease; GERD‐CC, gastroesophageal reflux disease with chronic cough.

### 3.2 Gastroesophageal reflux characteristics according to CC status

The patients in the GERD‐CC group had significantly higher reflux symptom scores than those in the GERD group (*P* = .007). Gastroscopy showed no esophageal erosion (NERD) in 25 patients (80.6%) in the GERD‐CC group and in 34 patients (72.3%) in the GERD group (Table 2). Five patients in GERD‐CC group had LA‐A and one LA‐B esophagitis, whereas six, two, three, and two patients in the GERD group had LA‐A, LA‐B, LA‐C, and LA‐D esophagitis, respectively.

**Table 2:** Reflux characteristics according to chronic cough status

	GERD‐CC group (n = 31)	GERD group (n = 47)	*P *value
Reflux symptom scores	6.4 ± 2.4	4.6 ± 3.1	.007
NERD (%)	80.6	72.3	.400
Esophageal acid reflux			
AET (min)	69.9 (30.6‐128.7)	27.7 (4.3‐78.9)	.030
>6% (%, n)	25.8% (8)	29.8% (14)	.702
4%‐6% (%, n)	32.3% (10)	17.0% (8)	.675
<4% (%, n)	41.9% (13)	53.2% (25)	.330
Episodes of acid reflux	39.0 (21.0‐61.0)	19.5 (7‐34.5)	.020
Episodes of prolonged acid reflux	2.0 (0‐5.0)	1.0 (0‐4.0)	.301
Longest reflux episode (min)	10.0 (4.8‐17.9)	6.8 (2.0‐13.3)	.102
DeMeester score	15.4 (6.1‐27.7)	7.1 (2.1‐17.0)	.070
Total episodes of reflux^a^	143.0 (104.0‐211.0)	103.0 (68.0‐141.0)	.008
Distal extent	111.0 (89.0‐162.8)	92.0 (52.8‐123.3)	.084
Proximal extent	15.0 (6.0‐28.5)	8.5 (5.0‐18.0)	.028
High extent	1.0 (0.75‐3.25)	1.0 (0‐2.25)	.385

Note

Data are presented as the median and interquartile range or percentage (number).

Abbreviations: AET, acid exposure time; GERD, gastroesophageal reflux disease; GERD‐CC, gastroesophageal reflux disease with chronic cough.

a All reflux episodes detected by impedance per patient.

Patients in the GERD‐CC group had significantly longer AET (*P* = .03), more frequent acid reflux episodes (*P* = .02) and higher DeMeester scores (*P* = .07) than those in the GERD group (Table 2). However, there was no significant difference in AET >6% (25.8% vs 29.8%), AET <4%, or AET 4%‐6% between the two groups (Table 2). Even though the rates of AET >6% were relatively low in both groups, more than 80 reflux episodes were detected by impedance in those patients with AET <6%, indicating that all enrolled patients had objective evidences of gastroesophageal reflux.

**Figure 3:**
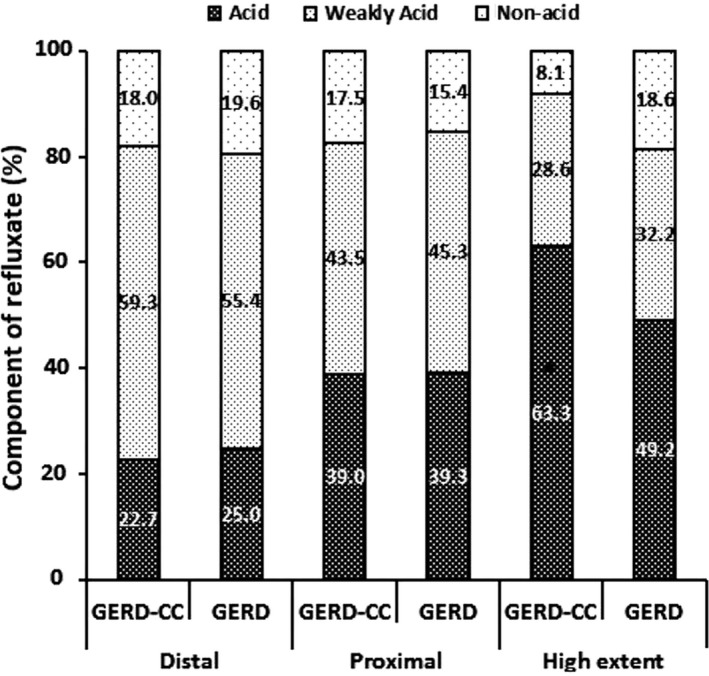
Component of refluxate according to reflux extent according to chronic cough status. GERD: gastroesophageal reflux disease, GERD‐CC: gastroesophageal reflux disease with chronic cough. * *P* < .05 compared with GERD group

According to pH‐impedance, patients in the GERD‐CC group had significantly more reflux episodes and proximal reflux episodes than those in the GERD group (both *P* < .05, Table 2). There were no significant differences in numbers of distal reflux (*P* = .084) or high‐reflux episodes between the two groups. The refluxates were acidic in 63.3% of high‐reflux episodes in the GERD‐CC group, which is significantly higher than 49.2% in the GERD group (*P* = .005, Figure 3). However, the percentage of acidity in distal and proximal reflux did not differ markedly between the two groups (*P* = .73 and *P* = .84, respectively).

### 3.3 Esophageal motility according to CC status

**Figure 4:**
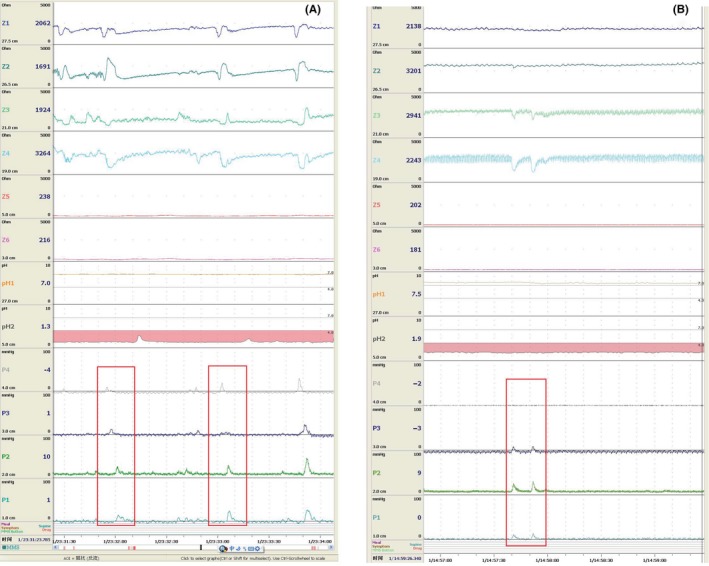
The ambulatory esophageal pH‐impedance‐pressure monitoring tracings show the low pan‐esophageal pressure of primary peristalsis (A) in a GERD‐CC patient and synchronous contraction of secondary peristalsis in a GERD patient (B). Abbreviations: GERD, gastroesophageal reflux disease; GERD‐CC, gastroesophageal reflux disease with chronic cough. Note: The typical low pan‐esophageal pressure waves and a synchronous contraction are marked with red frame

Synchronous ambulatory esophageal manometry detected 366 peristalsis waves during 78 prolonged acid reflux episodes. In the GERD‐CC group, 145 primary peristalsis and 67 secondary peristalsis waves occurred during the longest acid reflux episodes of each patient, whereas in the GERD group 110 primary peristalsis and 44 secondary peristalsis waves occurred during the longest acid episodes (Table 3). IEM, presenting as low pressure in both distal and proximal esophagus, occurred significantly more commonly during primary peristalsis (Figure 4A) in the GERD‐CC than in the GERD group (*P* < .001, Table 3), whereas low pressure in the distal esophagus was more common in the GERD group (*P* < .01). As for secondary peristalsis, IEM, mostly presenting as low pressure in the distal esophagus, was more common in the GERD than GERD‐CC group; however, in the GERD‐CC group, 63.9% of IEM presented as synchronous contraction (Figure 4B), significantly more frequently than in the GERD group (9.1%, *P* < .001).

**Table 3:** Features of ambulatory esophageal motility during prolonged acid exposure in GERD‐CC and GERD groups

	GERD‐CC group (n = 31)	GERD group (n = 47)	*P *value
Primary peristalsis	145	110	
Ineffective peristalsis (%)	104 (71.7%)	68 (61.8%)	.095
Distal low pressure (%)	28 (26.9%)	33 (48.5%)	.004
Proximal low pressure (%)	8 (7.7%)	3 (4.4%)	.390
Distal and proximal low pressure (%)	40 (38.5%)	8 (11.8%)	.000
Synchronous contraction (%)	28 (26.9%)	24 (35.3%)	.243
Secondary peristalsis	67	44	
Ineffective peristalsis (%)	47 (70.1%)	39 (88.6%)	.023
Distal low pressure (%)	17 (36.1%)	35 (90.9%)	.000
Proximal low pressure (%)	0	0	–
Synchronous contraction (%)	30 (63.9%)	4 (9.1%)	.000

### 3.4 Characteristics of reflux‐induced cough episodes

Monitoring of pH‐impedance‐pressure detected 206 coughing paroxysms, 126 (61.2%) of which occurred within two minutes of a reflux episode. Sixty‐three of these episodes were reflux‐cough episodes and 63 cough‐reflux episodes. All 63 reflux‐cough episodes occurred in 23/31 patients (74.2%) in the GERD‐CC group. Fourteen of the 23 patients with reflux‐cough episodes also had cough‐reflux episodes and 10 of these unrelated reflux and cough. Additionally, nine other also had unrelated reflux and cough. Three patients only had cough‐reflux episodes and five had no coughing paroxysms during the 24 hours of monitoring.

**Figure 5:**
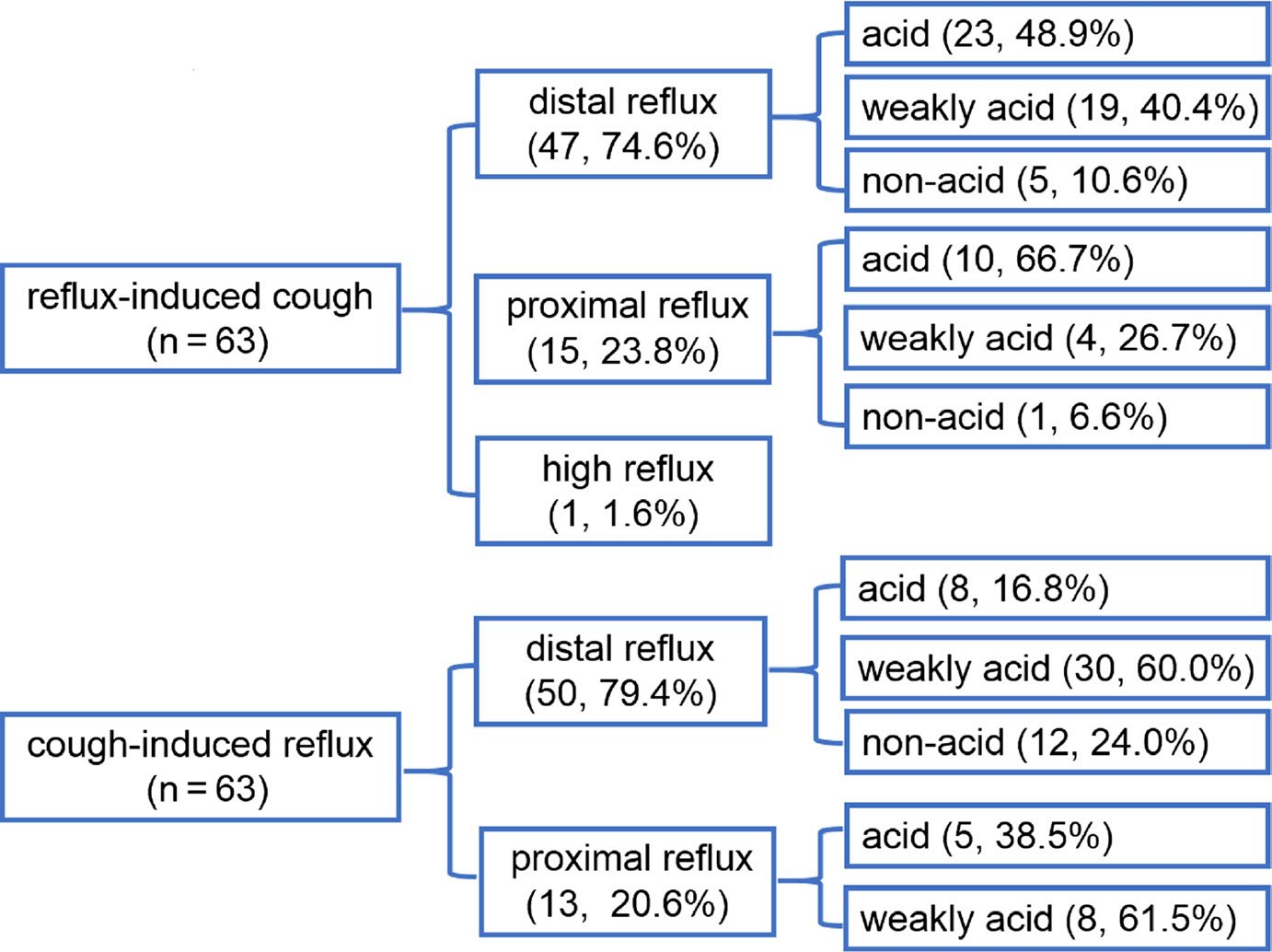
Characteristics of reflux in reflux‐induced cough and cough‐induced reflux episodes in patients with GERD‐CC. Note: n refers to the number of reflux‐induced coughing paroxysms or cough‐induced reflux episodes, not number of patients; values in the figures refer to the number of episodes and percentage (n, %)

Among 63 reflux‐cough episodes, 54.0% of the reflux episodes were acidic, 36.5% weakly acidic, and 9.5% non‐acidic. Furthermore, 74.6% reflux‐cough episodes resulted from distal extent reflux, 48.9% of these episodes being associated with acidic reflux, 40.4% with weakly acidic reflux, and 10.6% with non‐acidic reflux. Additionally, 23.8% reflux‐cough episodes resulted from proximal extent reflux, 66.7% of these episodes being associated with acidic reflux, 26.7% with weakly acidic reflux, and 6.6% with non‐acidic reflux. Only one reflux‐episode was associated with acidic high‐extent reflux (Figure 5).

Also, we monitored 16 reflux‐cough episodes in total of 823 proximal/high reflux while 47 reflux‐cough episodes in total of 4120 distal reflux (2.00% vs. 1.14%, *P* < .05).

### 3.5 Characteristics of cough‐induced reflux episodes

We found that 20.6% of the 63 cough‐reflux episodes that induced reflux were acidic reflux, 60.3% weakly acidic, and 19.1% non‐acidic. Furthermore, 79.4% reflux episodes were distal extent reflux, 60.0% of these being weakly acidic. Additionally, 20.6% episodes were proximal extent reflux, 61.5% of the refluxates being weakly acidic. No high‐extent reflux was recorded as associated with cough (Figure 5).

## 4 DISCUSSION

The relationship between gastroesophageal reflux and chronic cough is complex. Although there have been many studies and consensus has been reached on reflux‐cough syndrome, it is still difficult to diagnose reflux‐induced cough in an individual. Most previous studies^19^, ^20^, ^21^, ^22^ have focused on individuals with unexplained coughs and investigated the effects of reflux on cough. In contrast, we enrolled patients with GERD in our study and explored the differences between those with and without cough. We found that patients with GERD‐CC had more severe reflux episodes and proximal extent reflux than did GERD patients without CC, and that most high‐extent reflux is acidic. Proximal acid reflux and distal reflux jointly contribute to inducing reflux‐induced coughing in patients with GERD. Esophageal dysmotility, especially pan‐esophageal low pressure during primary peristalsis and synchronous contraction during secondary peristalsis, play important roles in GERD‐associated CC.

One important hypothesis concerning the mechanism of reflux‐induced cough is that proximal reflux and micro‐aspiration of gastric refluxate stimulate coughing by direct irritation of the respiratory tract. Previous studies have found that proximal acid reflux^30^ and aspiration of gastric contents (pepsin, bile acid, or lipid‐laden macrophages)^31^, ^32^, ^33^ can induce CC events. A recent study also found that volume clearance time and reflux burden play key roles in inducing coughing.^20^ However, other studies^7^, ^34^ found no difference between patients with CC and controls in proximal reflux events or bronchoalveolar lavage (BAL) pepsin or bile acids. Most studies have focused on the characteristics of reflux in patients with CC, whereas in our study we compared the characteristics of reflux in patients with GERD‐CC. A new finding of our study was that coughing paroxysms induced by proximal and high‐extent reflux occurred in 6/31 (19.4%) of patients with GERD‐CC and in 6/23 (26.1%) of reflux‐cough patients, most associated refluxate being acidic. We also found 16 reflux‐cough episodes in total of 823 proximal/high reflux, the percentage was higher than reflux‐cough episodes in distal reflux. These findings provide more detailed supportive evidence for acidic reflux of proximal and high‐extent inducing coughing in patients with GERD.

Another hypothesis for reflux‐cough episodes is stimulation of a vagal esophagobronchial reflex by reflux, triggering the cough reflex. Saline and acid infusion studies^35^, ^36^ have shown that cough frequency and amplitude are greater with acid than saline; infusion of acid into the esophagus increases cough sensitivity in patients with GERD and cough. Also, decreases in distal reflux and coughs after anti‐GERD therapy in patients with unexplained cough supports the distal‐reflux reflex mechanism.^37^ However, there is little evidence for transient distal reflux inducing cough or the associated reflux characteristics. We found distal reflux contributed to 74.6% of reflux‐cough episodes, the refluxate being acidic in almost half and weakly acidic in 40.4%. We also tried to compare additional characteristics of distal reflux that did or did not cause cough, but failed because of the huge difference in frequency of these episodes (47 distal refluxes causing cough vs. 3735 distal refluxes not causing cough).

Esophageal dysmotility is another important mechanism in GERD. Ineffective esophageal motility^38^, ^39^ and large breaks^10^, ^40^ are associated with reflux‐cough events. Meanwhile, long‐term exposure to acid is negatively correlated with esophageal body motility.^41^, ^42^ To the best of our knowledge, few studies have investigated esophageal motility during long‐term acid reflux in individuals with CC and GERD. We found that most primary and secondary peristalsis is ineffective (61.8%‐88.6%), low pressure amplitude in both the distal and proximal esophagus results in 38.5% IEM of primary peristalsis, and more synchronous contractions in secondary peristalsis result in IEM in patients with GERD‐CC. Those findings are consistent with impairment of primary and secondary peristalsis leading to ineffective esophageal clearance and prolonged exposure to acid.^43^, ^44^ Thus, we might extrapolate that low pan‐esophageal pressure amplitude in primary peristalsis and synchronous contraction in secondary peristalsis have important effects on reflux‐induced cough in patients with GERD‐CC.

Combining esophageal pH‐impedance and manometry monitoring is a proven diagnostic tool for identifying reflux and cough and guiding treatment of patients with reflux‐cough.^7^, ^19^, ^20^, ^21^, ^22^, ^45^, ^46^ We used a time window of two minute as indicating a temporal association between reflux episodes and coughing paroxysms.^7^, ^19^, ^20^, ^21^, ^22^, ^45^, ^46^ We found that 74.2% (23/31) of patients with GERD‐CC have reflux‐induced cough, 45.2% (14/31) having both reflux‐cough episodes and cough‐reflux episodes and 9.7% (3/31) having only cough‐reflux episodes. No coughing was recorded in 16.1% of patients with GERD‐CC. Distal reflux and weakly acidic reflux were more common in cough‐reflux events. The results of monitoring provided strong evidence that proton pump inhibitors (PPIs) are the optimal therapy for patients with GERD‐CC and cough caused by reflux, that is, adequate doses of more potent PPIs and prolonged treatment are indicated. As for reflux caused by cough, comprehensive antitussive measures might be more effective than overuse of PPIs whereas, if available, a prokinetic (ie, mosapride or prucalopride) may be indicated for patients with esophageal dysmotility (ie, IEM).^26^, ^47^

Our study has several limitations. Less than expected high‐extent reflux was detected, this discrepancy possibly being attributable to the high position of impedance loops (26 cm above the LES being defined as high extent in our study as compared with 15 cm above the LES being considered proximal, but not high‐extent reflux in a previous study).^30^ The highest pressure sensor was located 20 cm above LES, which could not detect swallows directly, so we used the impedance curve to identify the primary peristalsis. Another limitation was the relatively small sample size and small number of coughing paroxysms during which we recorded dynamic esophageal peristalsis. We only analyzed esophageal motility during the longest acid reflux episode of each patient, not during all prolonged episodes of long acid reflux. Moreover, we did not record and analyze self‐reports of cough symptom by patients; however, there have been some studies on the relationship between cough symptoms and reflux events.^14^, ^15^

## 5 CONCLUSIONS

In this study comparing patients with GERD with and without CC, we found that those with GERD‐CC had more severe reflux episodes and proximal extent reflux and that most high‐extent reflux was acidic. Proximal acid reflux and distal reflux‐reflex jointly contributed to occurrence of reflux‐induced cough in patients with GERD. Low pan‐esophageal pressure during primary peristalsis and synchronous contraction during secondary peristalsis during prolonged acid reflux play important roles in patients with GERD and CC. Thus, ambulatory pH‐impedance‐pressure monitoring may provide diagnostic and therapeutic evidence in patients who have failed PPI therapy, assisting optimization of PPI and/or indicating addition of prokinetic therapy in those with GERD‐CC and obvious dysmotilities, thus enhancing the integrated treatment in this subset of patients.

## CONFLICT OF INTEREST

No competing interests declared.

## AUTHOR CONTRIBUTIONS

XL and XF designed the study; SL and ZW performed pH‐impedance‐pressure monitoring; XL, HZ, XS, JL, DW, and XF enrolled patients and collected clinical data; XL and SL analyzed data and wrote the manuscripts; MK supervised and critically reviewed the manuscript; XF revised and finally approved the manuscript. All authors approved the final version of the manuscript. The abstract was presented at Digestive Disease Week, 2016 (Li X, Lin S, Wang Z, et al Gastroesophageal reflux disease and chronic cough: A possible mechanism elucidated by ambulatory pH‐impedance‐pressure monitoring. Gastroenterology 2016,150, 4 [Suppl 1]:S269).
